# Liver Stiffness by Transient Elastography Predicts Liver-Related Complications and Mortality in Patients with Chronic Liver Disease

**DOI:** 10.1371/journal.pone.0095776

**Published:** 2014-04-22

**Authors:** Jack X. Q. Pang, Scott Zimmer, Sophia Niu, Pam Crotty, Jenna Tracey, Faruq Pradhan, Abdel Aziz M. Shaheen, Carla S. Coffin, Steven J. Heitman, Gilaad G. Kaplan, Mark G. Swain, Robert P. Myers

**Affiliations:** 1 Liver Unit, Division of Gastroenterology and Hepatology, Department of Medicine, University of Calgary, Calgary, Alberta, Canada; 2 Department of Community Health Sciences, University of Calgary, Calgary, Alberta, Canada; 3 Medical Services, Alberta Health Services, Calgary, Alberta, Canada; Yonsei University College of Medicine, Republic of Korea

## Abstract

**Background:**

Liver stiffness measurement (LSM) by transient elastography (TE, FibroScan) is a validated method for noninvasively staging liver fibrosis. Most hepatic complications occur in patients with advanced fibrosis. Our objective was to determine the ability of LSM by TE to predict hepatic complications and mortality in a large cohort of patients with chronic liver disease.

**Methods:**

In consecutive adults who underwent LSM by TE between July 2008 and June 2011, we used Cox regression to determine the independent association between liver stiffness and death or hepatic complications (decompensation, hepatocellular carcinoma, and liver transplantation). The performance of LSM to predict complications was determined using the c-statistic.

**Results:**

Among 2,052 patients (median age 51 years, 65% with hepatitis B or C), 87 patients (4.2%) died or developed a hepatic complication during a median follow-up period of 15.6 months (interquartile range, 11.0–23.5 months). Patients with complications had higher median liver stiffness than those without complications (13.5 vs. 6.0 kPa; *P*<0.00005). The 2-year incidence rates of death or hepatic complications were 2.6%, 9%, 19%, and 34% in patients with liver stiffness <10, 10–19.9, 20–39.9, and ≥40 kPa, respectively (*P*<0.00005). After adjustment for potential confounders, liver stiffness by TE was an independent predictor of complications (hazard ratio [HR] 1.05 per kPa; 95% confidence interval [CI] 1.03–1.06). The c-statistic of liver-stiffness for predicting complications was 0.80 (95% CI 0.75–0.85). A liver stiffness below 20 kPa effectively excluded complications (specificity 93%, negative predictive value 97%); however, the positive predictive value of higher results was sub-optimal (20%).

**Conclusions:**

Liver stiffness by TE accurately predicts the risk of death or hepatic complications in patients with chronic liver disease. TE may facilitate the estimation of prognosis and guide management of these patients.

## Introduction

Liver fibrosis assessment is a vital aspect of the management of patients with chronic liver disease, both for guiding therapy and estimating prognosis. Most hepatic complications occur in patients with advanced fibrosis. Although liver biopsy had traditionally been used to stage fibrosis, it is limited by invasiveness, potential complications, cost, and difficulty of repetition for monitoring changes over time [1], [2]. Moreover, the accuracy of biopsy is influenced by subjectivity in histological interpretation and sampling error, which may lead to discordance in staging in up to 40% of patients [3]. In light of these limitations, noninvasive means for staging fibrosis have been developed including serum biomarkers (e.g. FibroTest [4], ELF Panel [5], FibroMeter [6]) and elastography (e.g. transient elastography [TE] [7]–[9] and magnetic resonance elastography) [10]. A wealth of literature has confirmed the accuracy of these tools for staging fibrosis [11] leading to their widespread use. More recently, attention has turned to the prognostic significance of these tools. Traditionally, the Child-Pugh score and Model for End-Stage Liver Disease (MELD) have been used to estimate prognosis in cirrhotic patients [12]; however, their utility in milder disease is unclear. On the contrary, emerging data suggest that in addition to the surrogate endpoint of fibrosis, the aforementioned noninvasive methods can predict mortality and liver-related complications. For example, the FibroTest is correlated with survival and has similar 5-year prognostic value to that of biopsy in patients with hepatitis B (HBV) [13], hepatitis C (HCV) [14], [15], and alcoholic liver disease [16]. Similar data has been reported for the ELF panel [17], AST-platelet ratio index (APRI) [18], FIB-4 [18], and TE (FibroScan; Echosens, Paris, France) [15], [19]–[24].

TE is an ultrasound-based tool for measuring liver stiffness as a surrogate of fibrosis that is widely used due to its high accuracy for the diagnosis of advanced fibrosis [25], [26]. Liver stiffness correlates with cirrhosis complications including variceal hemorrhage, ascites, and hepatocellular carcinoma (HCC) [27]. Many of these complications are portal hypertension-related; indeed, liver stiffness correlates with the hepatic venous pressure gradient [28], [29]. Furthermore, recent studies have shown an association between liver stiffness and survival. Among 1,457 patients with chronic HCV, Vergniol *et al.* reported that LSM by TE had superior diagnostic performance for predicting 5-year survival compared with biopsy [15]. In another study of patients with various conditions, Klibansky reported excellent diagnostic performance of TE for predicting a composite outcome including death, decompensation, and HCC (area under the receiver operating characteristic curve [AUROC], 0.87) [19].

The objective of our study was to examine the association between liver stiffness and the risk of death or liver-related complications in a large cohort of patients with diverse hepatic disorders and severities to reflect routine clinical practice. We report risk estimates at clinically relevant liver stiffness thresholds that that may help physicians estimate the prognosis of their patients and guide their management.

## Methods

### Study Population

This retrospective-prospective study included consecutive adults (≥18 years) with chronic liver disease (i.e. chronically elevated liver biochemistry and/or abnormal liver imaging) who underwent LSM by TE at the University of Calgary Liver Unit (UCLU) between July 2008 and June 2011. The UCLU is the major referral center for patients with liver disease in southern Alberta serving a catchment population of ∼1.5 million individuals. LSM by TE is performed routinely in all UCLU patients without overt evidence of hepatic decompensation. Patients with any of the following criteria were excluded: 1) non-residents of Alberta; 2) invalid provincial health numbers and/or the inability to link clinical information with administrative data (see below); 3) liver-related complications prior to or at the time of their FibroScan (see below); and 4) FibroScan failure, defined as no valid LSMs after ≥10 attempts. The Research Ethics Board at the University of Calgary approved the study protocol. The requirement for individual informed consent was waived for the study; however, patient records were anonymized and de-identified prior to analysis.

### Liver Stiffness Measurement

Two experienced operators performed all FibroScan examinations as per the manufacturer’s recommendations. Between July 2008 and July 2009, the M probe was used in all patients; thereafter, the XL probe was used in obese patients (body mass index [BMI]≥30 kg/m^2^). Briefly, with the patient lying in the dorsal decubitus position and the right arm in maximal abduction, the tip of the FibroScan transducer probe was placed on the skin between the ribs over the right lobe of the liver. Assisted by a sonographic image, a portion of the liver ≥6 cm thick and free of large vascular structures was identified and an attempt was made to collect ≥10 valid LSMs. The median liver stiffness value was considered representative of the elastic modulus of the liver. Fasting prior to the examination was not routinely required. As an indicator of variability, the ratio of the interquartile range (IQR) of liver stiffness to the median (IQR/M) was calculated. As recommended by Boursier and colleagues [30], examinations with median stiffness ≥7.1 kPa and an IQR/M >30% were considered poorly reliable [31]. Analysis of the results according to a prior definition of reliability (≥10 valid measurements, success rate ≥60%, and IQR/M ≤30%) [32] revealed similar results (data not shown).

### Administrative Data Sources

This study utilized three administrative databases with linkage using a unique identifier to identify the underlying liver disease etiologies, comorbidities, hepatic complications, and mortality of study participants. These databases have been used to examine the epidemiology [33], [34], outcomes [2], [35], [36], and coding accuracy [33], [37] of various medical conditions.

1. *Physician Claims Database*. This database includes claims submitted for payment by Alberta physicians for services provided to registrants of the Alberta Health Care Insurance Plan, a universal plan that covers over 99% of Alberta residents [38]. Each record includes the service provided, date, and up to three diagnosis fields. The database was queried from April 2001 to March 2011.
2. *Inpatient Discharge Abstract Database.* This database contains diagnosis, procedure, and mortality information on all discharges from Alberta hospitals. Chart validation studies have shown rates of agreement >95% for demographics and 75–96% for most responsible diagnosis codes [39]. The database was queried from January 1991 to January 2012.
3. *National Ambulatory Care Reporting System/Ambulatory Care Classification System Database.* This database contains information on facility-based ambulatory care including clinic and emergency department visits, same-day surgery, and day procedures [38]. The database was queried from July 1996 to December 2011.

### Outcomes and Predictor Variables

Patients were managed as per consensus recommendations and followed according to the discretion of their hepatologist. Our primary outcome measure was a composite that included overall mortality and hepatic complications, including features of decompensation (ascites, spontaneous bacterial peritonitis, jaundice, hepatic encephalopathy, variceal hemorrhage, hepatorenal syndrome), HCC, and liver transplantation. Complications were identified by querying the administrative databases for relevant diagnosis and procedure codes according to the *International Classification of Disease, Ninth Revision, Clinical Modification* (ICD-9-CM) [40], the *Tenth Revision of the ICD* (ICD-10) [41], the *Canadian Classification of Diagnostic, Therapeutic, and Surgical Procedures (CCP)* [42], and the *Canadian Classification of Health Interventions* [43] after the FibroScan examination (see Appendix S1). Since the administrative databases do not include data regarding the underlying cause of death, we could not examine liver-related mortality specifically.

The primary predictor variable was median liver stiffness examined as a continuous variable; according to established cut-offs (F0–1: stiffness <7.1 kPa; F2∶7.1–9.4 kPa; F3 (bridging fibrosis): 9.5–12.4 kPa; and F4 (cirrhosis): ≥12.5 kPa) [44]; and according to the following clinically-relevant thresholds: <10, 10–19.9, 20–39.9, and ≥40 kPa. Previous studies have used similar thresholds [15], [20], [45], [46]. Additional covariates included age, gender, the underlying liver disease, comorbidities, and FibroScan reliability [32]. Hepatic diagnoses were categorized according to a hierarchy as follows: HBV, HCV, autoimmune (including primary biliary cirrhosis, primary sclerosing cholangitis, and autoimmune hepatitis), hemochromatosis, alcoholic liver disease, nonalcoholic fatty liver disease (NAFLD), and other (see Appendix S1). For example, a patient with any diagnosis code for HBV with or without other hepatic diagnoses would be categorized as having HBV. Comorbidities occurring before the FibroScan were defined using the Elixhauser algorithm (liver diseases excluded), which has been validated in patients with hepatic and non-hepatic disorders [47], [48]. Based on literature describing an adverse relationship between several comorbidities and outcomes in this population (i.e. diabetes, renal failure, fluid and electrolyte disorders, coagulopathy, HIV/AIDS, alcohol and drug abuse, and malignancy) [48], we *a priori* examined these comorbidities individually and the remainder were categorized (as 0, 1, or ≥2). We also examined the type of FibroScan probe because the XL probe gives lower readings than the M probe [9]. Moreover, use of the XL probe was considered a surrogate marker for obesity since BMI is not available in the administrative data.

### Statistical Analyses

All analyses were performed using Stata v11.0 (StataCorp; College Station, TX). Between groups comparisons were made using Fisher’s exact, chi-square, and Mann-Whitney tests, as appropriate. Two-sided *P*-values <0.05 were considered statistically significant. The association between liver stiffness and hepatic complications or mortality was assessed using the Kaplan-Meier method and Cox proportional hazards regression [49]. The proportional hazards assumption was confirmed. Patients were followed from the date of their FibroScan until a complication or the end of follow-up of the administrative data (31 January 2012). Multivariate Cox models adjusted for age, gender, liver stiffness, FibroScan probe, and variables that were statistically significant in univariate analyses. Due to differences in liver stiffness measured using the XL and M probes [9], an interaction term between liver stiffness and probe was examined, but it was not statistically significant (data not shown).

To assess the performance of LSM by TE in predicting death or hepatic complications during follow-up, we calculated the sensitivity, specificity, accuracy, and positive (PPV) and negative predictive values (NPV) of a threshold of ≥20 kPa. Previous studies have shown that this threshold reliably excludes hepatic complications during medium-term follow-up [19], [21], [22]. Finally, to determine the ability of LSM to discriminate between patients who will and will not develop complications, the c-statistic with a modification for survival data was calculated. The c-statistic from the Cox model is conceptually analogous to the AUROC estimated for logistic models (1.0 indicates perfect discrimination and 0.5 is equivalent to chance) [50].

## Results

### Patient Characteristics and Liver Stiffness Results

**Figure 1:**
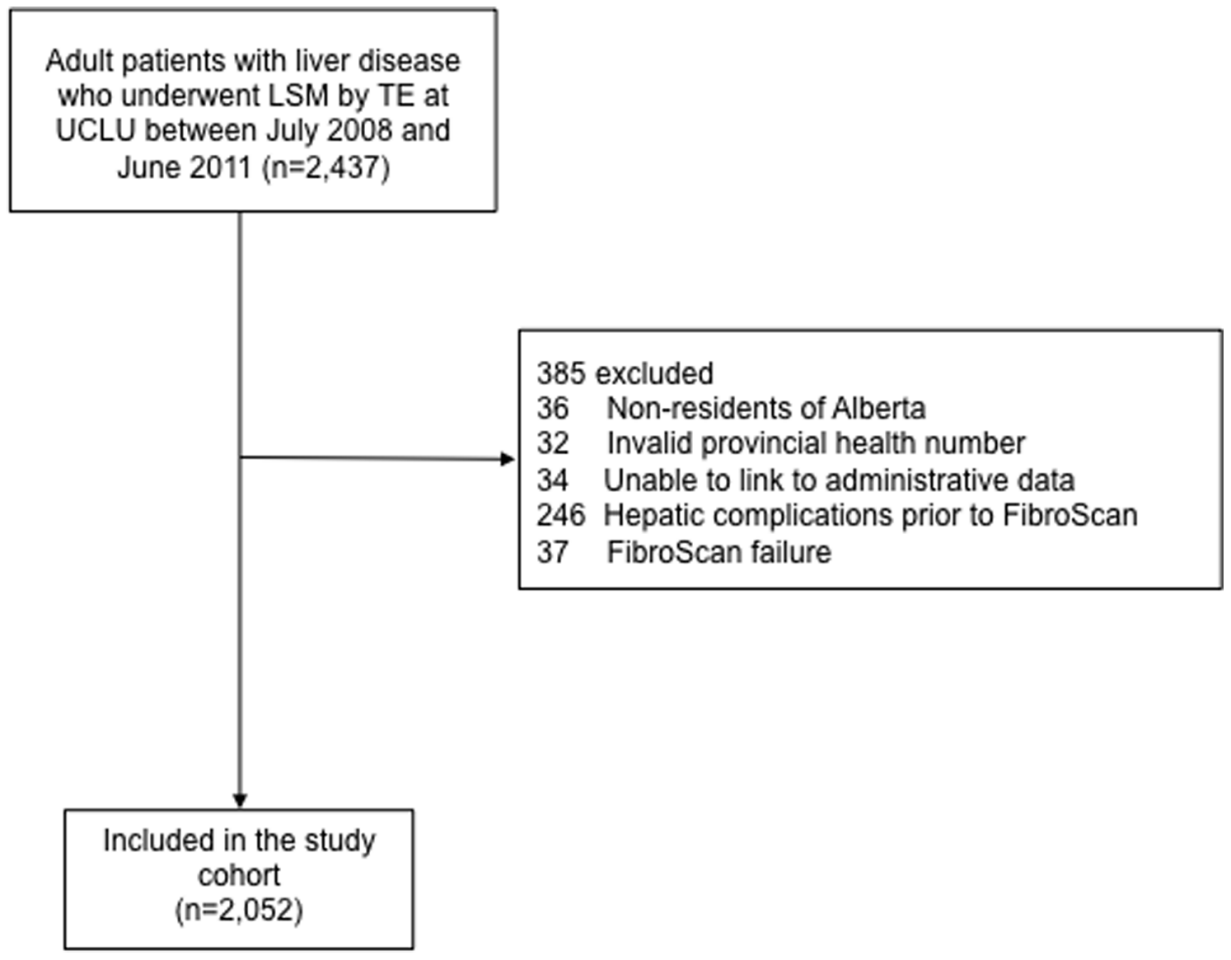
Flow diagram of study participants.

In total, 2,437 patients had a LSM by TE during the study period. After excluding 385 ineligible patients, 2,052 were included in the study cohort (Figure 1 and Table 1). The median age was 51 years (IQR 40–58) and 55% were male. The majority had HCV (36%) or HBV (29%), while 7% had NAFLD, 5% had autoimmune liver disease, 3% had hemochromatosis, and 2% had alcoholic liver disease. FibroScans were performed using the M probe in the majority (87%) of patients; 4.5% (n = 93) were poorly reliable. The median liver stiffness value was 6.1 kPa (range, 2.3–75 kPa; IQR 4.6–9.0 kPa). According to cut-offs recommended by Castera *et al.* [44], an estimated 61% of patients had F0–1 fibrosis, 15% had F2, 8% had F3, and 15% had F4 (cirrhosis).

**Table 1:** Characteristics of the Study Cohort According to the Occurrence of Hepatic Complications or Mortality During Follow-Up.

Variable	Total Cohort*(n = 2,052)	Without Complications(n = 1,965)	With Complications*(n = 87)	Unadjusted Hazard Ratio(95% CI)
**Male**	55% (1,134)	55% (1,081)	61% (53)	1.23 (0.80–1.89)
**Age**, *years*	50 (40–58)	50 (40–57)	55 (46–60)	**1.03 (1.01–10.5)**
**Hepatic diagnosis**				
Hepatitis B	29% (588)	29% (569)	22% (19)	0.85 (0.42–1.73)
Hepatitis C	36% (736)	36% (699)	43% (37)	1.30 (0.69–2.46)
Autoimmune	5.0% (102)	4.9% (96)	6.9% (6)	1.75 (0.66–4.60)
Hemochromatosis	3.0% (61)	3.0% (60)	1.2% (1)	0.44 (0.06–3.40)
Alcohol	2.2% (45)	2.2% (40)	5.8% (5)	**3.41 (1.22–9.57)**
NAFLD	6.8% (140)	6.8% (134)	6.9% (6)	1.16 (0.44–3.04)
Other/unknown	19% (380)	19% (367)	15% (13)	Ref
**Comorbidities**				
Diabetes mellitus	10% (205)	9.4% (184)	24% (21)	**3.04 (1.87–4.99)**
Renal failure	3.9% (80)	3.7% (72)	9.2% (8)	**2.69 (1.30–5.56)**
Fluid/electrolyte disorders	12% (238)	11% (221)	20% (17)	**1.94 (1.14–3.30)**
Coagulopathy	6.0% (123)	5.3% (105)	21% (18)	**4.21 (2.51–7.08)**
Malignancy †	26% (542)	26% (516)	30% (26)	1.11 (0.70–1.76)
Alcohol abuse	10% (208)	10% (198)	11% (10)	1.22 (0.63–2.35)
Drug abuse	9.4% (192)	9.0% (177)	17% (15)	**2.15 (1.23–3.75)**
HIV	1.0% (21)	1.0% (20)	1.2% (1)	0.95 (0.13–6.81)
Other comorbidities				
0	28% (580)	28% (559)	24% (21)	Ref
1	29% (590)	29% (567)	26% (23)	1.07 (0.59–1.93)
≥2	43% (882)	43% (839)	49% (43)	1.38 (0.82–2.32)
**Liver stiffness**, *kPa*				
Median (IQR)	6.1 (4.6–9.0)	6.0 (4.6–8.8)	13.5 (7.8–29.9)	**1.05 (1.04–1.06)**
F0–1 (<7.1 kPa)	61% (1,253)	63% (1,235)	21% (18)	Ref
F2 (7.1–9.4 kPa)	15% (316)	15% (305)	13% (11)	**2.46 (1.16–5.22)**
F3 (9.5–12.4 kPa)	8.2% (168)	8.0% (156)	14% (12)	**4.98 (2.40–10.3)**
F4 (≥12.5 kPa)	15% (315)	14% (269)	53% (46)	**10.71 (6.21–18.48)**
**M probe (vs. XL probe)**	87% (1,788)	88% (1,720)	78% (68)	**0.47 (0.28–0.79)**
**Poorly reliable liver stiffness measurement**	4.5% (93)	4.1% (81)	14% (12)	**3.21 (1.74–5.91)**

Data are median (IQR) or proportions (% [n]). Hazard ratios in bold are statistically significant (*P*<0.05).

*Complications include hepatic decompensation, HCC, liver transplantation, or death.

† Malignancy includes lymphoma, solid tumors without metastases, and metastatic cancer.

### Predictors of Hepatic Complications and Mortality

During a median follow-up period of 15.6 months (IQR 11.0–23.5), 87 patients (4.2%) died or developed a hepatic complication, corresponding to an overall incidence of 2.8 cases per 100 person-years (95% confidence interval [CI] 2.3–3.5). In the subpopulation of 315 patients with presumed cirrhosis, 46 cases (14.6%) developed a complication, corresponding to an overall incidence of 10.1 (95% CI 7.5–13.4) per 100 person-years (vs. 1.6 [95% CI 1.1–2.1] per 100 person-years in 1,737 non-cirrhotic patients; *P*<0.00005). Of the 124 total complications, specific events are outlined in Table 2. The median intervals between LSM and a complication, including transplantation (n = 5) specifically, were 5.7 months (IQR 1.5–10.6) and 4.1 months (IQR 0.7–8.4), respectively.

**Table 2:** Specific Hepatic Complications and Mortality (n = 87)*.

Complication	% (n)
**Hepatic decompensation**	3.3% (67)
Variceal hemorrhage	2.0% (40)
Ascites	1.3% (26)
Hepatic encephalopathy	0.5% (11)
Jaundice	0.3% (6)
Hepatorenal syndrome	0.2% (3)
Spontaneous bacterial peritonitis	0.05% (1)
**HCC**	0.7% (15)
**Liver transplantation**	0.2% (5)
**Death**	0.8% (17)

*Individual patients could have multiple complications; hence the total (n = 124) exceeds 87 patients.

**Figure 2:**
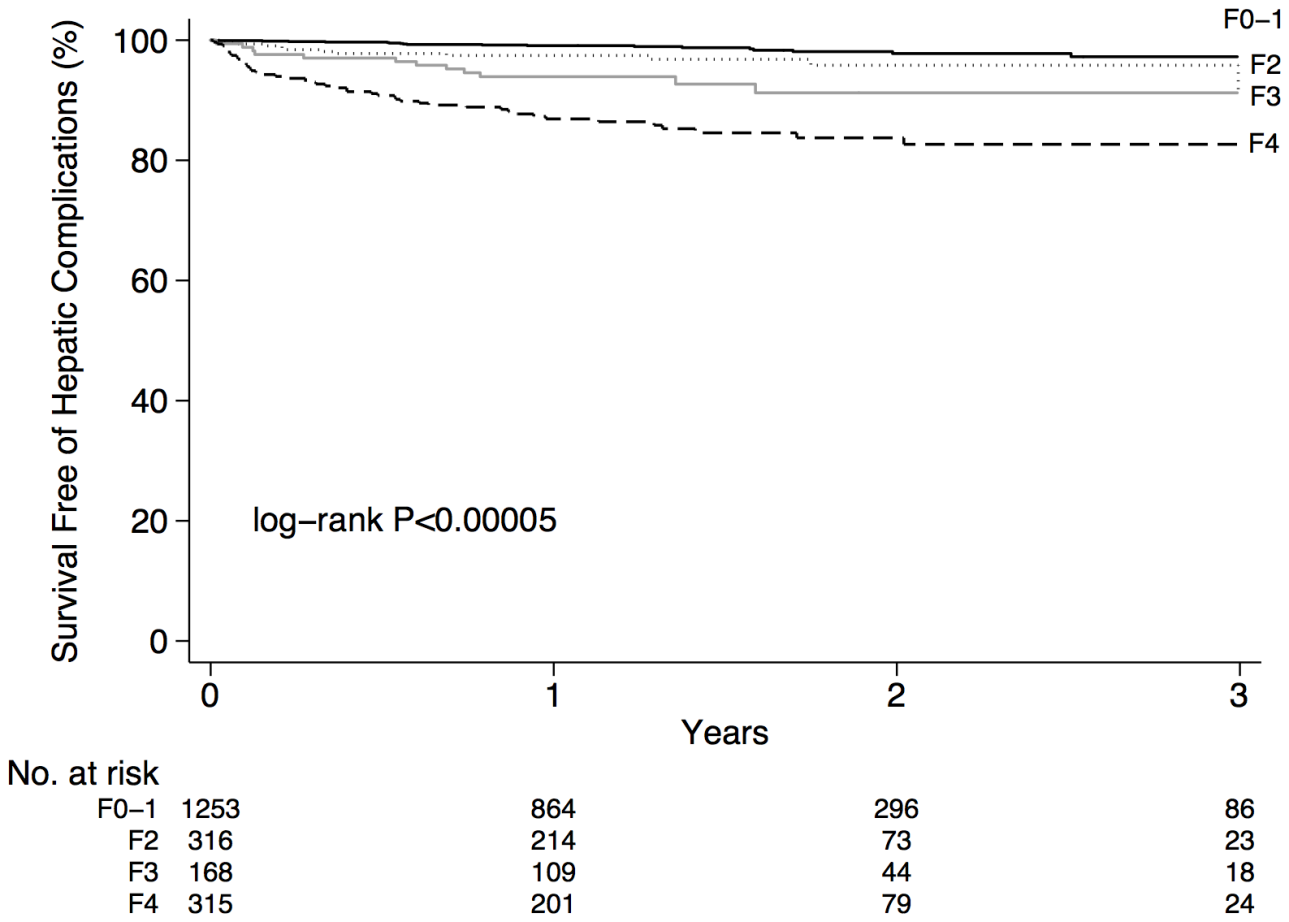
Unadjusted survival free of hepatic complications according to LSM by TE categorized as F0–1 (liver stiffness <7.1 kPa), F2 (7.1–9.4 kPa), F3 (9.5–12.4 kPa), and F4 (cirrhosis; ≥12.5 kPa).

**Figure 3:**
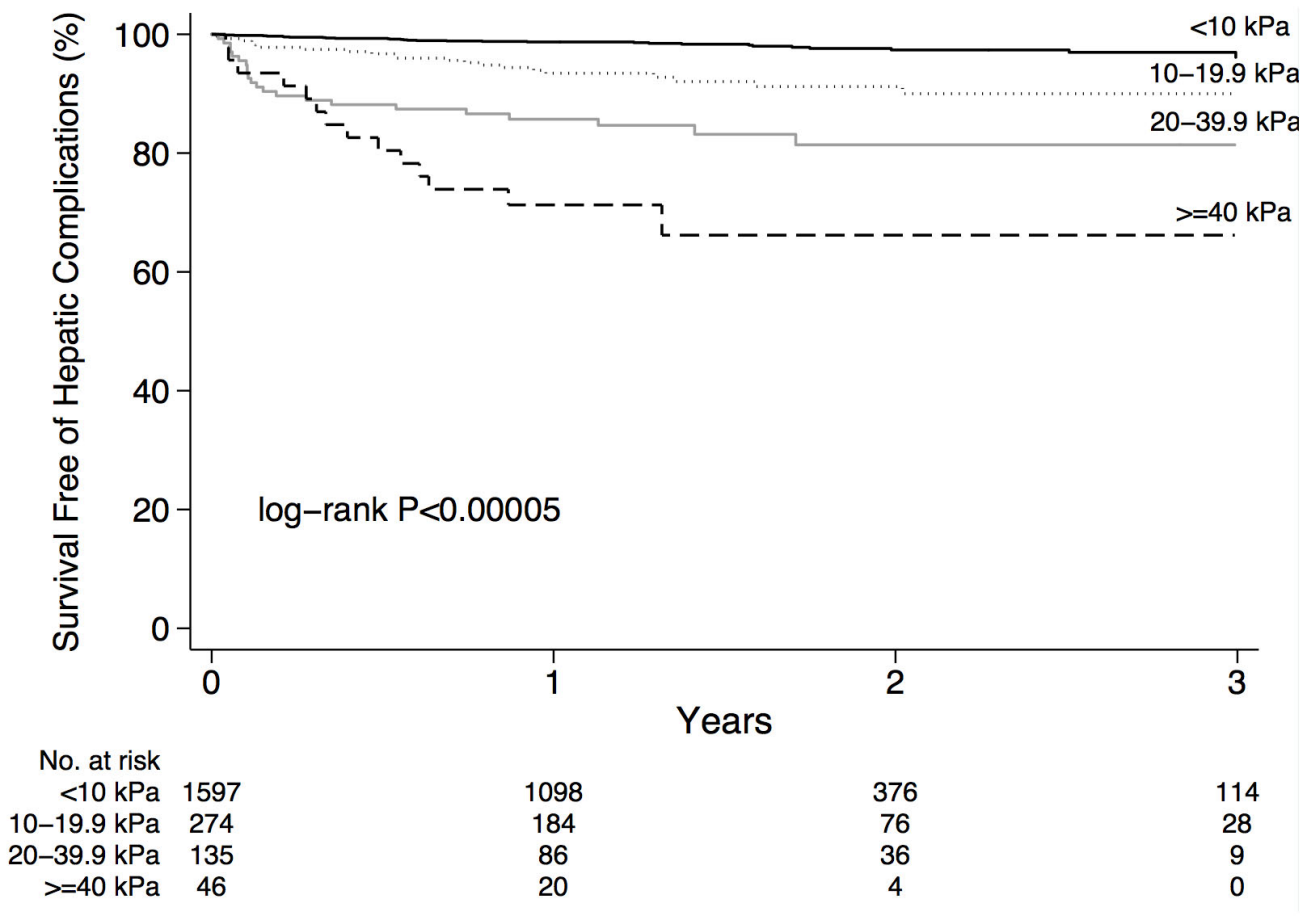
Unadjusted survival free of hepatic complications according to LSM by TE categorized as <10 kPa, 10–19.9 kPa, 20–39.9 kPa, and ≥40 kPa.

Patients who died or developed a hepatic complication during follow-up had higher median liver stiffness than patients without complications (13.5 kPa vs. 6.0 kPa; *P*<0.00005; Table 1). Survival free of complications was reduced in patients with an estimated fibrosis stage of F2, F3, or F4 compared with F0–1 (*P*<0.00005; Figure 2). Similarly, the risk of complications increased with liver stiffness within the advanced range (*P*<0.00005; Figure 3). Specifically, the overall incidence rates of death or hepatic complications in patients with liver stiffness <10 (n = 1,597), 10–19.9 (n = 274), 20–39.9 (n = 135), and ≥40 kPa (n = 46) were 1.23 (95% CI 0.9–1.8), 4.9 (3.2–7.5), 11.3 (7.4–17.1), and 29.0 (17.2–49.0) cases per 100 person-years, respectively (*P*<0.00005). In patients with liver stiffness <10, 10–19.9, 20–39.9, and ≥40 kPa, actuarial rates of death or hepatic complications at 1, 2 and 3 years were 1.3% (95% CI 0.8–2.0%), 2.6% (1.7–4.0%) and 3.9% (2.2–6.7%); 6.6% (4.1–10.4%), 8.8% (5.6–13.6%) and 10% (6.4–15.6%); 14% (9.4–22%), 19% (12–28%) and 19% (12–28%); and 29% (18–44%), 34% (21–52%) and 34% (21–52%), respectively (*P*<0.00005).

After adjustment for age, gender, hepatic diagnosis, comorbidities, and FibroScan probe and reliability, liver stiffness was an independent predictor of hepatic complications or mortality (Table 3). Specifically, the adjusted risk of complications increased 5% per 1-kPa increase in liver stiffness (hazard ratio [HR] 1.05; 95% CI 1.03–1.06). In subgroup analyses according to liver disease etiology, liver stiffness was predictive of complications in patients with HBV (HR 1.04; 95% CI 1.00–1.08), HCV (HR 1.06; 1.04–1.07), and other conditions (HR 1.05; 1.03–1.06). Additional predictors included diabetes (HR 1.79; 95% CI 1.02–3.14) and coagulopathy (HR 2.20; 1.27–3.81). In supplemental analyses in which liver stiffness was categorized rather than examined as a continuous variable, similar results were obtained (Table 3). For example, compared with patients with liver stiffness below 10 kPa, the risk of complications increased 3-fold in those with liver stiffness from 10 to 19.9 kPa (HR 3.22; 95% CI 1.81–5.75), 7-fold between 20 and 39.9 kPa (HR 7.02; 3.88–12.7), and 12-fold with liver stiffness ≥40 kPa (HR 12.5; 6.21–25.0).

**Table 3:** Multivariate Analysis of Predictors of Hepatic Complications and Mortality (n = 2,052)*.

Variable	Hazard Ratio (95% CI)	*P*-value
Age, *per year*	1.01 (0.99–1.03)	0.16
Male gender	1.10 (0.71–1.70)	0.68
Alcoholic liver disease	1.03 (0.39–2.69)	0.95
**Diabetes mellitus**	**1.79 (1.02–3.14)**	**0.04**
Renal failure	1.66 (0.74–3.71)	0.22
Fluid and electolyte disorders	1.16 (0.65–2.07)	0.63
**Coagulopathy**	**2.20 (1.27–3.81)**	**0.005**
Drug abuse	1.71 (0.96–3.06)	0.07
FibroScan probe (M vs. XL)	0.81 (0.47–1.40)	0.45
Poorly reliable liver stiffness measurement	1.58 (0.84–2.99)	0.16
**Liver stiffness**		
**Per 1-kPa increase**	**1.05 (1.03–1.06)**	**<0.0005**
**F0–1 (<7.1 kPa)**	Ref	Ref
**F2 (7.1–9.4 kPa)**	**2.16 (1.01–4.64)**	**0.05**
**F3 (9.5–12.4 kPa)**	**4.23 (2.01–8.90)**	**<0.0005**
**F4 (≥12.5 kPa)**	**7.64 (4.22–13.8)**	**<0.0005**
**<10 kPa**	Ref	Ref
**10–19.9 kPa**	**3.22 (1.81–5.75)**	**<0.0005**
**20–39.9 kPa**	**7.02 (3.88–12.7)**	**<0.0005**
**≥40 kPa**	**12.5 (6.21–25.0)**	**<0.0005**

*Hazard ratios for all variables (except those listed for specific liver stiffness categories) were obtained from a model including liver stiffness examined as a continuous variable.

### Performance of Liver Stiffness for Predicting Complications

**Figure 4:**
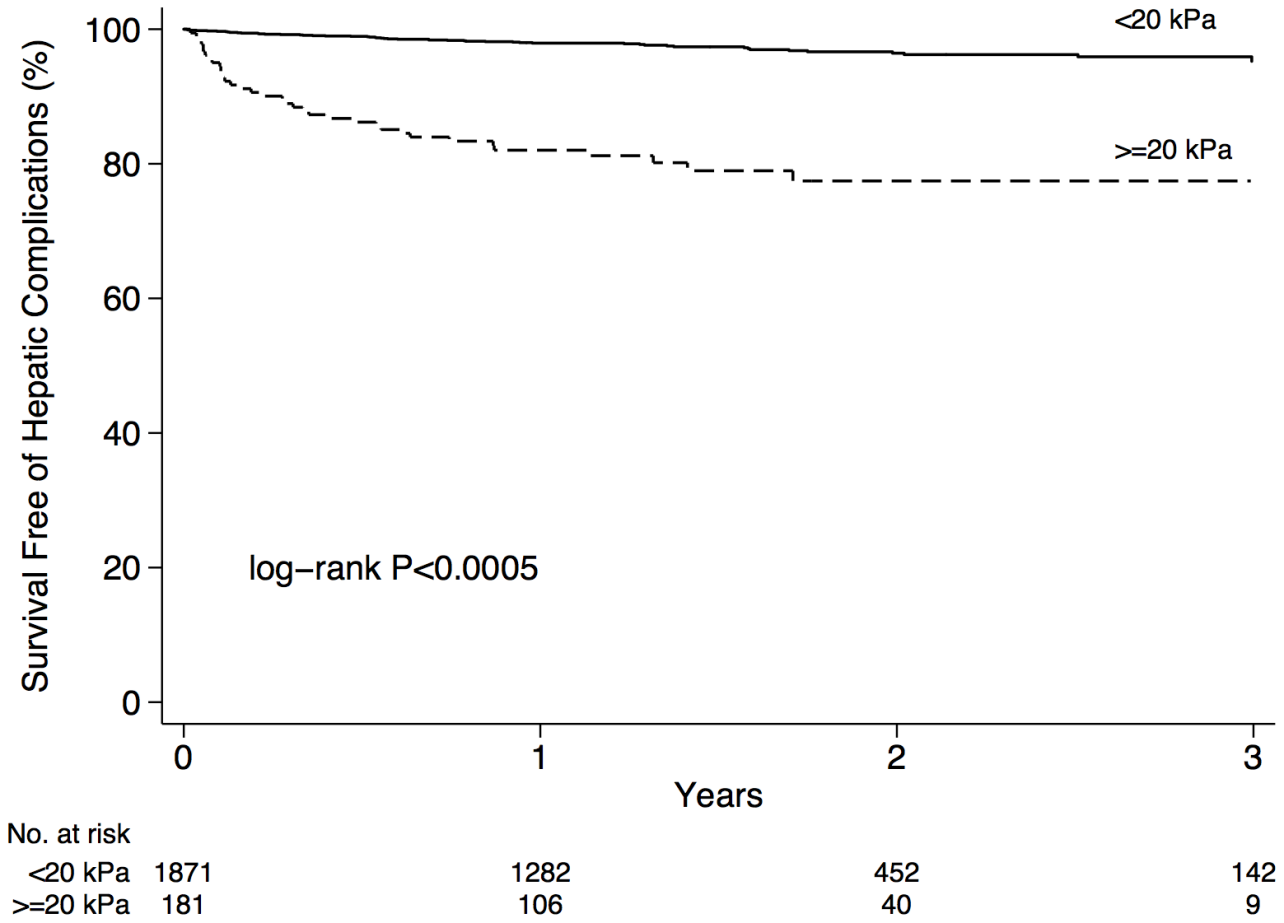
Unadjusted survival free of hepatic complications according to the 20-threshold for liver stiffness.

The c-statistic of liver-stiffness for the prediction of hepatic complications and mortality was 0.80 (95% CI 0.75–0.85). The sensitivity, specificity, PPV, NPV, and accuracy of a liver stiffness value ≥20 kPa for predicting complications were 41% (95% CI 31–52%), 93% (91–94%), 20% (14–26%), 97% (96–98%), and 90% (89–92%), respectively. The actuarial rates of death or hepatic complications at 3 years in patients with liver stiffness ≥20 kPa and <20 kPa were 23% (95% CI 16–30%) and 4.7% (3.2–7.0%), respectively (Figure 4; *P*<0.0005). For the secondary outcomes of hepatic decompensation or HCC (n = 76) and overall mortality (n = 17), the c-statistics of liver stiffness were 0.82 (95% CI 0.77–0.87) and 0.67 (0.55–0.80), respectively.

## Discussion

This is the largest study to examine the prognostic significance of liver stiffness by TE. In over 2,000 patients with various liver disease etiologies, we demonstrate that liver stiffness is an independent predictor of hepatic complications and mortality after adjustment for age, gender, underlying disease, comorbidities, and other potential confounders. Specifically, for every 1-kPa increase in liver stiffness, the adjusted risk of complications increased 5%. Importantly, we have generated risk estimates stratified according to clinically relevant stiffness categories that may help physicians to estimate the prognosis of their patients and guide their management. Specifically, the 3-year actuarial rates of death or hepatic complications in patients with liver stiffness <10, 10 to <20, 20 to <40, and ≥40 kPa were 3.9%, 10%, 19%, and 34%, respectively (Figure 3). Corresponding incidence rates of complications were 1.2, 4.9, 11.3, and 29.0 cases per 100 person-years, respectively.

These data are consistent with other reports. For example, in patients with HCV, Vergniol and colleagues reported that the 5-year risk of death increased from 4% in patients with liver stiffness ≤9.5 kPa, to 34% with stiffness >20 kPa and 53% with stiffness >40 kPa [15]. In another study of HIV/HCV-coinfected, cirrhotic subjects, the risk of death or HCC at 3 years was 19% in patients with liver stiffness <40 kPa compared with 37% among those with higher values [20]. The event rates of complications that we report according to liver stiffness are clinically relevant, particularly since the proportion of patients undergoing biopsy is declining as use of TE increases. Our data suggest that in patients with stiffness >10 kPa, and particularly those with stiffness >20 kPa, enhanced follow-up to monitor for complications is advisable. On the contrary, patients with liver stiffness <10 kPa can be safely reassured that their risk during several years of follow-up is low (<5%). As the burden of liver disease increases (e.g. due to NAFLD), the ability to defer close surveillance in patients with low liver stiffness values will be important for providing reassurance and minimizing healthcare expenditures.

The stepwise increase in risk of complications that we observed according to liver stiffness categories within the cirrhotic range (Figure 3) suggests that TE offers prognostic information above and beyond that provided by liver biopsy. Whereas the stage of cirrhosis is traditionally defined by histological evidence of regenerative nodules with one or two qualitative categories (e.g. METAVIR stage 4 or Ishak stages 5 and 6), the dynamic range of LSM is much greater. Since the quantity of fibrous tissue deposition varies widely in cirrhosis, it is clear that the risk of complications is not uniform among cirrhotic patients. In this regard, the ability to express liver stiffness as a continuous variable (from ∼12.5 to 75 kPa in cirrhosis) or in an arbitrary number of categories represents an advantage for prognostication compared with biopsy. Indeed, several studies have shown better performance of FibroScan (and other non-invasive tools) compared with biopsy for predicting hepatic complications [15], [19]. For example, in the study of Vergniol *et al*. [15], the AUROCs for 5-year survival of FibroScan, FibroTest, and biopsy were 0.82, 0.80, and 0.76, respectively. The AUROC observed for TE (0.80) in our study is consistent with these reports, and supports its excellent discriminatory ability. At a threshold liver stiffness value ≥20 kPa, TE had a sensitivity, specificity, and accuracy of 41%, 93%, and 90%, respectively. Liver stiffness values <20 kPa effectively exclude complications (NPV 97%; Figure 4); however, the PPV of higher results (20%) does not allow one to adequately identify which patients will go on to develop a complication. The latter patients should perhaps undergo enhanced follow-up.

Our study includes several additional findings worthy of discussion. First, we identified an increased risk of complications among patients with liver stiffness corresponding to each of F2, F3, and F4 fibrosis compared with F0–1 fibrosis (Figure 2). For example, patients with liver stiffness between 7.1 and 9.4 kPa (∼F2) at baseline had a two-fold risk of complications compared with those with lower liver stiffness (F0–1; Table 3). Since progression to cirrhosis over this time frame in a patient with F2 fibrosis is unexpected, we hypothesize that this relates to underestimation of fibrosis by TE in some cases. This is not unexpected since the sensitivity of a liver stiffness ≥9.5 kPa for advanced fibrosis (F3–F4) is only 73% [44]. Second, our data suggest that the influence of liver stiffness on complications is independent of which FibroScan probe is used. A unique aspect of our study is the inclusion of patients scanned using the XL probe, not available in most prior studies. Confirmation of this association is important because liver stiffness measured using the XL probe is typically 1–2 kPa lower than with the M probe [9]. Also, we considered the XL probe a surrogate marker for obesity, which we could not reliably identify using our databases. Surprisingly, this was not a significant predictor of complications, although the study may have been underpowered to detect this association. On the contrary, diabetes and coagulopathy were independently associated with complications. Diabetes is an important risk factor for all-cause mortality in general, plus the progression of chronic liver diseases including HBV, HCV, and NAFLD [51]. The association between coagulopathy and liver-related complications is largely due to recorded diagnoses of thrombocytopenia in the administrative data, although some patients had hereditary and acquired coagulation defects (data not shown). Although we attempted to exclude patients with hepatic decompensation prior to their FibroScan, it is possible that some patients were coagulopathic, yet clinically compensated. Nevertheless, exclusion of the 123 patients with coagulopathy did not influence the association between liver stiffness and complications (data not shown).

Our study has several limitations. First, our findings rely on the validity of the administrative databases that we used. Although chart abstraction studies have confirmed their accuracy for many hepatic [33], [36], [37] and non-hepatic [52] conditions, additional validation is necessary. Likewise, it is possible that patients developed a complication (e.g. mild ascites), yet did not seek medical attention or had a medical visit without capture of the diagnosis in the administrative data. Second, the databases lack laboratory data to confirm the diagnosis and assess disease severity (e.g. with the Child-Pugh or MELD scores). Prior studies have suggested that the discrimination of liver stiffness can be improved by considering additional laboratory variables. For example, Klibansky *et al*. reported that a composite score including TE, AST/ALT ratio, and MELD outperformed TE for predicting clinical outcomes (AUROCs 0.93 vs. 0.86) [19]. In addition, since liver biochemistry is not available in the administrative databases, we cannot exclude an impact of non-fibrotic histologic features impacting liver stiffness (e.g. marked ALT elevation due to severe hepatic inflammation). Similarly, information regarding alcohol consumption, obesity, dyslipidemia, and specific treatments (e.g. antiviral therapy) that can have disease-modifying effects are not available or poorly coded in the administrative databases. Nevertheless, it is unlikely that over the restricted follow-up in our study that these factors would have had a significant impact on the clinical course of most patients. Moreover, a prior study in patients with HCV showed that the prognostic significance of liver stiffness persists after adjustment for antiviral treatment [15]. Third, we considered liver stiffness cut-offs validated for HCV despite the diverse etiologies of liver disease in our cohort. We chose this approach for simplicity despite literature showing that these thresholds may be disease-dependent. Finally, our study is limited by a relatively small number of events that precluded an analysis of the associations between liver stiffness and individual complications. Nevertheless, other studies have shown a correlation between liver stiffness and sequelae including death, HCC, variceal hemorrhage, and hepatic insufficiency following surgical resection [15], [19]–[24], [53].

In conclusion, liver stiffness measured by TE is an independent predictor of hepatic complications and mortality in patients with chronic liver disease. The risk estimates that we report at clinically relevant liver stiffness cut-offs will provide valuable information to physicians and assist them in counseling their patients regarding their prognosis and may help guide their follow-up.

## Supporting Information

Appendix S1 **Diagnosis and procedure codes used to define hepatic diagnoses and hepatic complications.**
(DOCX)
