# Supplementary material for: Liver Stiffness by Transient Elastography Predicts Liver-Related Complications and Mortality in Patients with Chronic Liver Disease
Source: PLoS One. 2014 Apr 22;9(4):e95776. doi: 10.1371/journal.pone.0095776 (PMC3995722; doi:10.1371/journal.pone.0095776)
Supplement: Appendix S1 — Diagnosis and procedure codes used to define hepatic diagnoses and hepatic complications. (DOCX) [file pone.0095776.s001.docx]

**APPENDIX S1**

**Diagnosis and procedure codes used to define hepatic diagnoses and hepatic complications**

| **Diagnoses** | **ICD-9-CM Diagnosis Codes ^42^** | **ICD-10 Diagnosis**  **Codes ^43^** | **Procedure Codes**  **(ICD-9-CM,^42^ CCI,^45^ CCP^44^)** |
| --- | --- | --- | --- |
| ***Hepatic Diagnoses*** |  |  |  |
| Hepatitis B | 070.2, 070.3 | B16, B18.0, B18.1, B19.1 | --- |
| Hepatitis C | 070.41, 070.44, 070.51, 070.54, 070.7 | B17.1, B18.2, B19.2 | --- |
| Non-alcoholic fatty liver disease | 571.8 | K75.81, K76.0 | --- |
| Alcoholic liver disease | 571.0, 571.1, 571.2, 571.3 | K70 | --- |
| Primary biliary cirrhosis | 571.6 | K74.3 | --- |
| Primary sclerosing cholangitis | 576.1 | K83.0 | --- |
| Autoimmune hepatitis | 571.42 | K75.4 | --- |
| Hemochromatosis | 275.0 | E83.1 | --- |
|  |  |  |  |
| ***Hepatic Complications*** |  |  |  |
| Jaundice | 782.4 | R17 | --- |
| Ascites | 789.5 | R18 | 54.91, 1.OT.52.^^, 66.91 |
| Spontaneous bacterial peritonitis | 567.2 | K65 | --- |
| Variceal hemorrhage | 456.0, 456.20 | I85.0, I85.01, I85.11, I98.20, I98.3 | 44.91, 44.93, 42.91, 42.33, 1.NA.13.^^, 54.91, 56.91, 10.06 |
| Hepatorenal syndrome | 572.4 | K76.7 | --- |
| Hepatic encephalopathy | 572.2 | K72 | --- |
| Hepatocellular carcinoma | 155.0 | C22.0 | --- |
| Liver transplantation | 996.82, V42.7 | T86.4, Z94.4 | 50.5, 1.OA.85.^^ |

CCI, Canadian Classification of Interventions; CCP, Canadian Classification of Diagnostic, Therapeutic, and Surgical Procedures; ICD, International Classification of Diseases.
